# Case 3/2017 - A 47-Year-Old Female with Refractory Heart Failure and Embolic Acute Myocardial Infarction

**DOI:** 10.5935/abc.20170106

**Published:** 2017-07

**Authors:** João Gabriel Batista Lage, Paulo Sampaio Gutierrez

**Affiliations:** Instituto do Coração (Incor) HC-FMUSP, São Paulo, SP - Brazil

**Keywords:** Heart failure, Myocardial Infarction, Thromboembolism

The patient was a 47-year-old white single female referred for medical treatment to the Instituto do Coração, born and coming from the state of São Paulo, with four children, unemployed, but reporting having worked in coffee farming in inner Minas Gerais state.

At the time of her first medical consultation (September 25, 2015), she reported dyspnea on minimal exertion, orthopnea and anasarca, which had started 2 months earlier. She denied angina pectoris, previous myocardial infarction and syncope. She knew she had systemic arterial hypertension and diabetes mellitus, and was being treated at a basic health unit. She used to smoke (20 packs/year), but quit the habit 2 months before. She denied consuming alcoholic beverages and illicit drugs, as well as a family history of cardiovascular disease.

She was on metformin (2550 mg/day), furosemide (80 mg/day), carvedilol (12.5 mg/day), and losartan (50 mg/day).

On her first medical consultation, her physical exam showed regular general condition and dyspnea in the horizontal position. Her blood pressure was 100/70 mmHg, and heart rate, 102 bpm. Her pulmonary auscultation revealed no respiratory sound on the base of the right lung and no rales. Her cardiac auscultation showed regular gallop rhythm, due to the presence of the third heart sound, and no murmur. Her abdomen was globose, tense, painless, with signs of huge ascites. Her extremities were cold and edematous (++/4+), with symmetrical pulses.

The electrocardiogram on the medical consultation showed sinus rhythm, heart rate of 97 bpm, left atrial overload and indirect signs of right atrial overload (Peñaloza-Tranchesi sign), low voltage of the QRS complexes in the frontal plane and no progression of the R wave in V_1_ to V_4_ (probable electrically inactive area in the anterior wall), and diffuse changes of ventricular repolarization (Figure 1).

**Figure 1:**
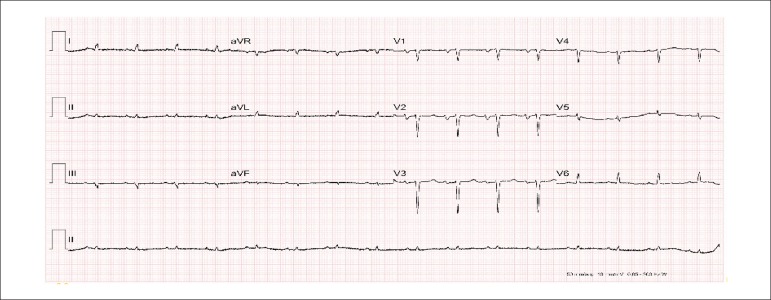
ECG: left atrial overload and indirect signs of right atrial overload Peñaloza-Tranchesi sign), low voltage of the QRS complexes in the frontal plane and no progression of the R wave in V_1_ to V_4_ (probable electrically inactive area in the anterior wall), and diffuse changes of ventricular repolarization.

Her chest X-ray showed bilateral veiling of costophrenic sinus, with pleural effusion up to half of the right hemithorax, normal aorta and global heart enlargement (++++/4+) (Figure 2).

**Figure 2:**
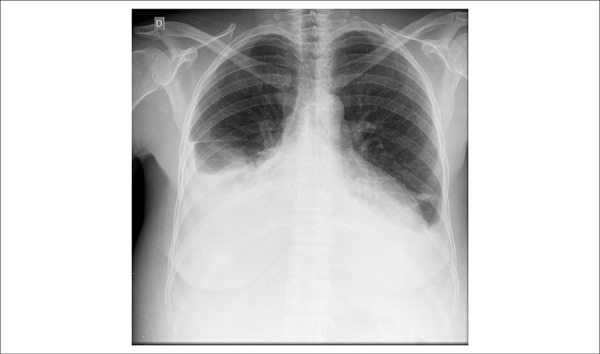
Chest X-ray showing bilateral veiling of costophrenic sinus, with pleural effusion up to half of the right hemithorax, normal aorta and global heart enlargement (++++/4+).

Her laboratory tests were as follows: hemoglobin 14.8 g/dL; hematocrit 46%; leukocytes 9950/mm^3^; creatinine 0.87 mg/dL; sodium 141 mg/dL; potassium 4.3 mg/dL; and negative serology for Chagas disease.

On that medical consultation, spironolactone 25 mg was added, and, due to gastrointestinal intolerance, metformin was replaced by glicazide 30 mg/day.

Returning to medical consultation at the outpatient clinic (December 16th, 2015), she reported marked improvement of the dyspnea, then triggered only by large exertion. Her physical exam revealed no pathological jugular venous distention, blood pressure of 100/70 mmHg, heart rate of 100 bpm. Her pulmonary auscultation showed no respiratory sound on the base of the right lung. Her cardiac auscultation evidenced the presence of the third heart sound and no murmur. Her abdomen was globose, tense, painless, with liver palpable 3 cm from the right costal margin. Her lower limbs had mild edema (+/4+). She had not undergone the tests requested on her first medical consultation. Her prescription was then changed: carvedilol to metoprolol 50 mg/day.

After missing her subsequent medical consultation, the patient was hospitalized on July 25, 2016, due to heart failure decompensation. She had mixed shock, with decreased level of awareness and increased levels of myocardial injury markers. She required dobutamine for hemodynamic control. Empiric antibiotic therapy was initiated with ceftriaxone and clarithromycin, being the patient later submitted to orotracheal intubation for respiratory support.

Bedside chest X-ray on that day evidenced bilateral veiling of costophrenic sinus, with reduced transparency of the lower third of the right hemithorax (pleural effusion), increased pulmonary vascular bed with Kerley’s B lines, and marked heart enlargement (Figure 3).

**Figure 3:**
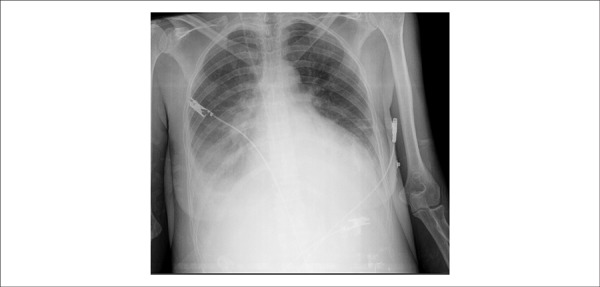
Bedside chest X-ray showing bilateral veiling of costophrenic sinus, with pleural effusion up to half of the right hemithorax, normal aorta and global heart enlargement (++++/4+).

Her laboratory tests revealed: hemoglobin 14.1 g/dL; hematocrit 44%; leukocytes 15100/mm^3^ (band neutrophils 6%, segmented neutrophils 84%, lymphocytes 5%, and monocytes 5%); platelets 195000/mm^3^; CK-MB 9.7 ng/mL; troponin I 0.654 ng/mL; ALT 42 U/L; AST 80 U/L; urea 87 mg/dL; creatinine 1.18 mg/dL; sodium 132 mEq/L; potassium 3.5 mEq/L; International Normalized Ratio (INR) 2.3; ratio between activated thromboplastin times 1.06; magnesium 1.6 mEq/L; total bilirubin 3.14 mg/dL; direct bilirubin 2.19 mg/dL; C-reactive protein 156.50 mg/L; arterial lactate 22 mg/dL. Arterial blood gas analysis (with oxygen therapy) showed pH of 7.52, pCO_2_ of 29.6 mmHg, pO_2_ of 176 mmHg, oxygen saturation of 99.9%, bicarbonate of 24.1 mmol/L, and base excess of 2.2 mmol/L.

Bedside transthoracic echocardiography showed: left ventricular diffuse hypokinesia and ejection fraction of 20%; marked right ventricular hypokinesia; marked mitral and tricuspid regurgitation and poor leaflet coaptation; pulmonary valve with signs of pulmonary hypertension; mild pericardial effusion and presence of large heterogeneous mass in the left ventricle, measuring 30x28 mm, compatible with intracavitary thrombus. Estimated systolic pulmonary artery pressure of 65 mmHg.

Coronary angiography revealed coronary arteries without proximal lesions, but the anterior interventricular branch showed a 95% distal lesion, and the diagonal branch showed distal occlusion. Neither the circumflex artery nor the right coronary artery showed any sign of obstruction (Figures 4A, 4B, 4C, 4D).

**Figure 4:**
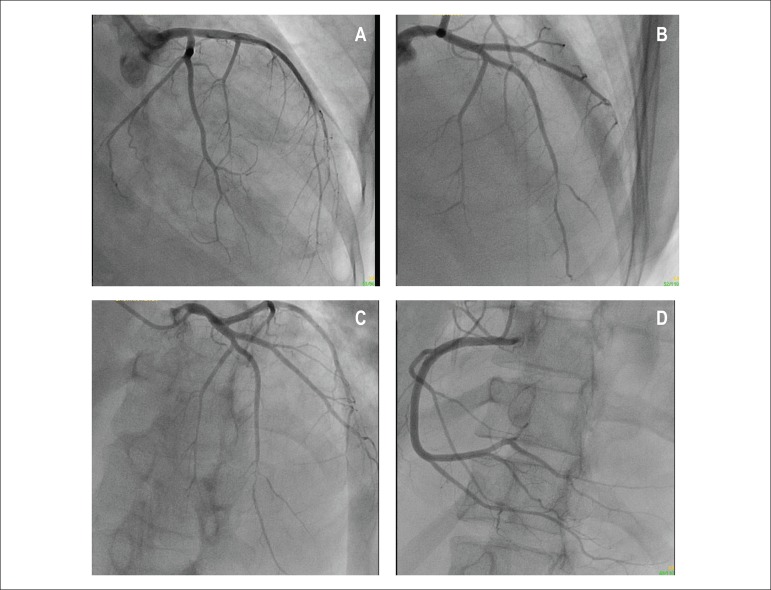
Coronary angiography: A) caudal RAO: 95% distal lesion in the anterior descending branch and occlusion of the diagonal branch; B) cranial RAO: 95% distal lesion and distal occlusion of the diagonal branch; C) cranial LAO: 95% distal lesion in the anterior descending branch; D) LAO: right coronary artery with no lesion.

The hypothesis of infarction of embolic cause was raised. The patient was referred to the intensive care unit, with progressive increase of vasoactive drugs and later introduction of noradrenaline and widening of the antimicrobial spectrum to meropenem and vancomycin. The patient had refractory shock and died on July 27, 2016, with multiple organ dysfunction.

## Clinical aspects

This case can be approached in two ways: chronic disease and acute decompensation. Taking the chronic disease way, some possible etiologies of heart failure can be considered. With the negative Chagas serology and her known comorbidities, the major hypotheses to be considered for this patient are hypertensive heart disease (dilated phase), microcirculation disease due to diabetes mellitus, and idiopathic dilated cardiomyopathy.^1-3^ The patient attended to only two medical consultations, being her complementary investigation unfinished. Now, taking the acute decompensation way, it was relatively clear at the beginning that the infectious hypothesis was the most plausible, being the elevation in the levels of myocardial necrosis markers probably related to sepsis and hemodynamic instability (type 2 acute myocardial infarction). However, after the results of the other complementary tests (echocardiography and coronary angiography), the hypothesis of acute myocardial infarction of embolic cause gained strength, mainly due to the finding of an intracavitary thrombus on the first exam. Therefore, we hypothesize that the significant left ventricular dysfunction determined the formation of the thrombus, whose fragment embolized to the coronary circulation, causing an acute myocardial infarction, culminating with dysfunction worsening, thus triggering the cascade that led to the patient’s death.

Some of the causes of coronary emboli are heart valvular disease, cardiomyopathy, coronary atherosclerosis and atrial fibrillation. In a postmortem study by Prizel et al.,^4^ an intracavitary thrombus was present in 33% of the cases. Nevertheless, a superimposed infectious cause for decompensation cannot be ruled out. **(João Gabriel Batista Lage, MD)**

**Diagnostic hypothesis**: syndromic: heart failure due to heart disease with left ventricular ejection fraction reduction; etiological: dilated cardiomyopathy; final: acute myocardial infarction due to thromboembolism to the coronary arteries and cardiogenic shock. **(João Gabriel Batista Lage, MD)**

## Postmortem examination

**Figure 5:**
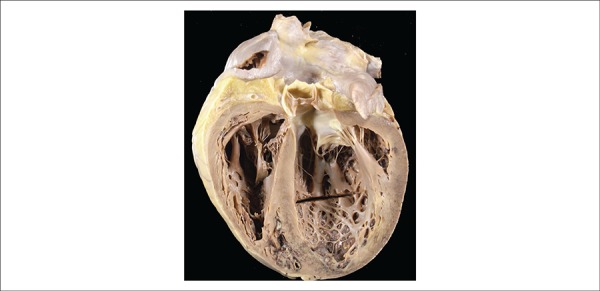
Longitudinal section of the heart showing dilatation of the cavities.

The heart showed global dilatation of the four chambers (Figure 5), and no significant changes in the valves and coronary arteries. The microscopic exam showed neither inflammatory infiltrate nor any type of deposit, and the muscle fibers were thin and had enlarged nuclei, denoting hypertrophy. Thrombi were present in the tips of both ventricles (Figure 5). The diagnosis of systemic arterial hypertension was based only on information provided by the patient, and there was no renal arteriolosclerosis. In the lack of genetic study, thus, neither decompensated hypertensive cardiomyopathy nor idiopathic dilated cardiomyopathy can be diagnosed for sure, the latter seeming more likely.

**Figure 6:**
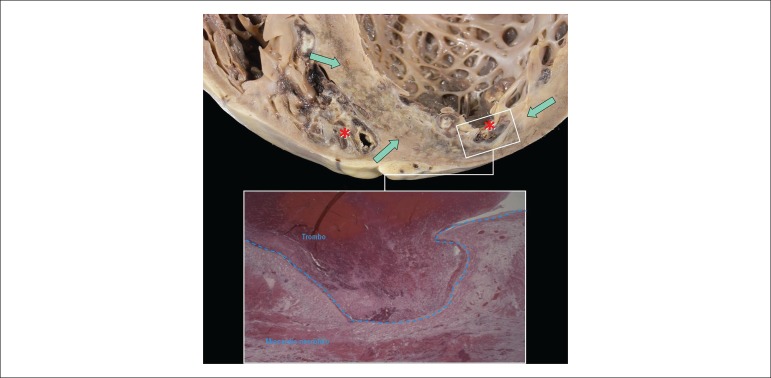
Longitudinal section of the apex of the heart showing thrombi in both ventricles (asterisks) and myocardial infarction (arrows).

**Figure 7:**
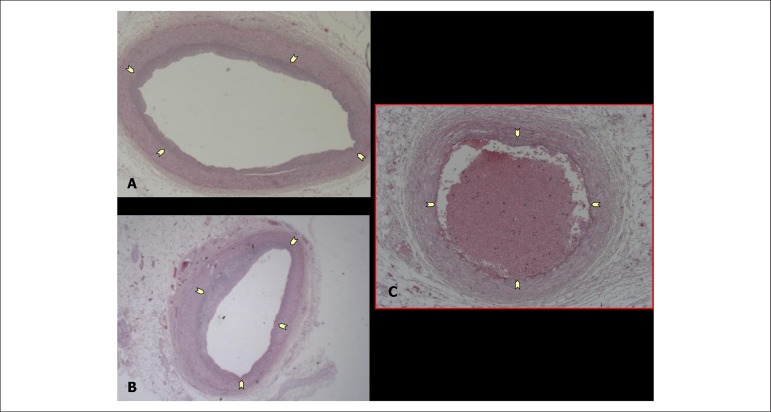
Cross-sectional histological sections of segments of the coronary arteries. A and B- right coronary artery and left main coronary artery, respectively, showing intima layer (delimited by the arrows) without signifcant obstructions; C- the 6^th^ centimeter of the anterior interventricular branch (anterior descending) occluded with a thrombus-embolus.

There was myocardial infarction of approximately 2 weeks, affecting the apical region of the left ventricular anterior and septal walls (Figures 5 and 6). On microscopic exam, the coronary arteries were normal or had minimal intimal lesions (Figures 7A and 7B). On the 6^th^ centimeter of the anterior interventricular branch (anterior descending), there was lumen occlusion by a material with characteristics of thrombus-embolus (Figure 7C).

**Figure 8:**
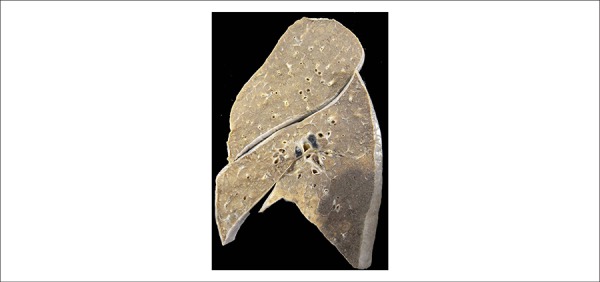
Gross section of the right lung showing hemorrhagic infarction in the lower lobe (darker triangular area).

In the lower lobes of the lungs, there were infarctions, small to the left and large to the right (Figure 8), which were considered the final factor triggering death.

In the other organs, there were changes resulting from congestive heart failure, with chronic passive congestion, general visceral congestion, anasarca and cachexia. **(Paulo Sampaio Gutierrez, MD)**

**Major disease**: idiopathic dilated cardiomyopathy.

**Cause of death**: pulmonary thromboembolism **(Paulo Sampaio Gutierrez, MD)**

## Comments

It is worth noting, in this patient with dilated cardiomyopathy, the presence of myocardial infarction, in whose region, there was mural thrombus in both the right and left ventricles. The major issue is the cause of the infarction. The coronary arteries, on both coronary angiography and morphological exam, had no significant atherosclerotic disease, except for distal embolization of the anterior interventricular branch (anterior descending). Adding the coronary angiographic finding with the presence of infarction, one might consider that the later resulted from embolization to a coronary artery. The myocardial infarction and later that of the lung might have been caused by embolism from the ventricular thrombi. It is worth noting that, coincidentally, the infarction happened in the same area of the thrombus originating it. Another possibility might be the infarction resulting from another process, such as vasospasm, generating thrombi, which caused the terminal embolism.

Although there are other similar cases in the literature,^5,6^ the appearance of transmural infarction in patients with idiopathic dilated cardiomyopathy is uncommon. **(Paulo Sampaio Gutierrez, MD).**

**Section editor:** Alfredo José Mansur (ajmansur@incor.usp.br)

**Associated editors:** Desidério Favarato (dclfavarato@incor.usp.br)

Vera Demarchi Aiello (vera.aiello@incor.usp.br)
